# 1434. Analysis of hand hygiene techniques using the artificial intelligence (AI)-based analysis tool SCORE!

**DOI:** 10.1093/ofid/ofad500.1271

**Published:** 2023-11-27

**Authors:** Hiroshi Kakeya, Yasuyo Okada, Akiko Fujita, Kiyotaka Nakaie, Waki Imoto, Wataru Shibata, Koichi Yamada

**Affiliations:** Osaka Metropolitan University, Osaka, Osaka, Japan; Osaka Metropolitan University Hospital, Osaka, Osaka, Japan; Osaka Metropolitan University Hospital, Osaka, Osaka, Japan; Osaka Metropolitan University Hospital, Osaka, Osaka, Japan; Osaka Metropolitan University, Osaka, Osaka, Japan; Osaka Metropolitan University, Osaka, Osaka, Japan; Osaka Metropolitan University, Osaka, Osaka, Japan

## Abstract

**Background:**

Hand hygiene is the most important technique for controlling nosocomial infections. While quantitative evaluations based on the amount of hand sanitizer used and qualitative evaluations such as compliance rates based on direct observation are commonly used to evaluate the effectiveness of this education, more objective evaluation indicators are needed. Here, we evaluated the effectiveness of hand hygiene education by scoring hand hygiene techniques using an artificial intelligence (AI)-based analysis tool.

**Methods:**

Hand disinfection (proper spreading of fluorescent paint) and hand washing (proper rinsing of fluorescent paint) techniques were scored before and after a hand hygiene education video training using the AI-based analysis tool SCORE!Ⓡ(Moraine Corporation, Tokyo).

Images were taken with an iPhone before and after hand disinfection and washing. The image data were divided into 72 parts, including the wrists and the backs of the right and left palms. Hand hygiene techniques for each part were evaluated using AI. The effectiveness of the training was also evaluated by job categories.

**Results:**

The pre- and post-education scores of 935 participants (128 physicians, 484 nurses, 56 nursing assistants, 93 other medical staff, and 174 administrative staff) increased for hand disinfection (P < 0.01) and decreased for hand washing (P < 0.01). The scores for hand disinfection increased for all occupations, while those for hand washing decreased for all occupations except nursing assistants, indicating that hand disinfection was highly effective in education but hand washing was less effective. In a comparison by job categories, there was a large difference in scores between the job categories in terms of the highest and lowest scores for both hand disinfection and hand washing. In the site-specific evaluation, the back of the hand scored higher in hand disinfection, with no significant difference between left and right hands. On the other hand, the palm and left hand scored higher in hand washing, differing from the hand disinfection results.

**Conclusion:**

Evaluating hand disinfection scores using “SCORE!” allows for the assessment of even minor changes that may be difficult to detect visually and provides information on individual techniques that may require improvement.

**Disclosures:**

**Hiroshi Kakeya, MD, PhD**, Asahi Kasei Pharma Corporation: Honoraria|Merck Sharp & Dohme: Honoraria|Sumitomo Pharma Co., Ltd.: Honoraria

